# Identification of differentially expressed genes in microarray data in a principal component space

**DOI:** 10.1186/2193-1801-2-60

**Published:** 2013-02-19

**Authors:** Luis Ospina, Liliana López-Kleine

**Affiliations:** Departamento de Estadística, Universidad Nacional de Colombia, Ciudad Universitaria carrera 30 con calle 45, edificio 404, Bogotá, Colombia

**Keywords:** C_d_ closeness measure, Differentially expressed genes, Microarray data, Principal component analysis

## Abstract

Microarray experiments are often conducted in order to compare gene expression between two conditions. Tests to detected mean differential expression of genes between conditions are conducted applying correction for multiple testing. Seldom, relationships between gene expression and microarray conditions are investigated in a multivariate approach. Here we propose determining the relationship between genes and conditions using a Principal Component Analysis (PCA) space and classifying genes to one of two biological conditions based on their position relative to a direction on the PC space representing each condition.

## Introduction

Since the introduction of microarrays in the nineties, several methods have been developed for their analysis and especially for the detection of differentially expressed genes (Dudoit et al., 2002). The analysis of diverse microarray data sets has allowed a better understanding of biological phenomena at the molecular level. Nowadays, more and more microarray experiments are available and multivariate data analysisareneeded to process them and extract useful biological knowledge. Multivariate methods are useful for this task, as they allow reducing dimensions and revealing data structure (Lebart et al., 1995), which means that individuals and variables can be separated on a principal component space. Nevertheless, microarray data often appear to be very noisy and descriptive multivariate methods do not allow extracting enough knowledge from the data to explain biological phenomena (Dudoit et al., 2002). Due to this limitation, univariate methods concentrating on each gene at a time are used to detect differentially expressed genes (Tusher et al., 2001). These methods account for the context of high dimensions and high variability by applying multiple test corrections at different levels of hypothesis testing. Nevertheless, the multivariate context and possible emerging properties are lost (López-Kleine et al, 2012).

One multivariate method, that accounts for high dimension and improves data structure detection by combining dimension reduction and prediction is Discriminant Analysis on Principal Components (DAPC) (Jombart et al., 2010). This method applies a Discriminant Analysis (DA) on the principal component space (PCA) in order to separate individuals in populations without having an *a prior*i on the data structure. Grouping needed for DA is obtained previously by k-means. Moreover, this method allows a probabilistic assignment of individuals to the different groups that are obtained. Initially, the authors have applied DAPC to Single Nucleotide Polymorphism (SNP) allele frequency data.

As for SNPs data, in which each SNP on the genome can be seen as a new variable, microarray data can be understood as profiles: each row (gene) represents an individual and the column values generate the expression profiles through microarray experiments (here: replicates of two conditions). Data are intensity measures of messenger RNA present for each gene at the given biological condition. So, multivariate methods will be used to reveal position of individuals (genes in this case) relative to the replicates of biological conditions (variables). A multivariate method that could deal with the mentioned noise and the multivariate structure of microarray data is the Principal Component Analysis. Once microarray condition replicates (variables) are reduced to a smaller number of PCs, each replicate can be represented through a linear combination on the PC space and the closeness of genes to each biological condition can be determined taking into account the factorial map of variables obtained through the PCA.

The aim of this work was to propose a closeness measure C_d_ that allows determining genes that belong to each one of two biological conditions, each of which is characterized by a direction D on the PC space. This methodology can be extended to more than two conditions because on a principal component space several groups of variables could be detected and classification of genes to more than one group can be undertaken applying the same methodology. The obtained belonging to a condition is comparable to a classification into a group obtained by DAPC. We also compared classifications combining these two methods. We applied these methodologies to real and simulated data and conclude that the best results are obtained for C_d_or the combination of both methodologies and that the performance depends on the data structure.

## Materials and methods

### Data

Microarray data sets were obtained from the Tomato Expression Database website (http://ted.bti.cornell.edu/). In this study we used experiments that were carried out using the TOM1 DNA chip. For the differential expression analysis we focused on the experiments carried out by Christine Smart and collaborators (Accession number E022: http://ted.bti.cornell.edu/cgi-bin/TFGD/miame/experiment.cgi?ID=E022) where gene expression profiling of infection of tomato *Phytophthora infestans* in the field was studied. The goal of this experiment was to gain insight into the molecular basis of the compatible interaction between *P. infestans* and its hosts. We used the data from that experiment in order to apply the new C_d_ closeness measure or the previously described DAPC or a combination of both for detecting genes that were differentially expressed in *P. infestans* inoculated plants vs. non inoculated plants in the field. For this comparison four time points were available at 0, 12, 36 and 60 hours with 8 replicates of each condition. For analysis here we focused on the 16 experiments available for the last time point (60 hours after inoculation).

### C_d_ closeness measure

**Figure 1:**
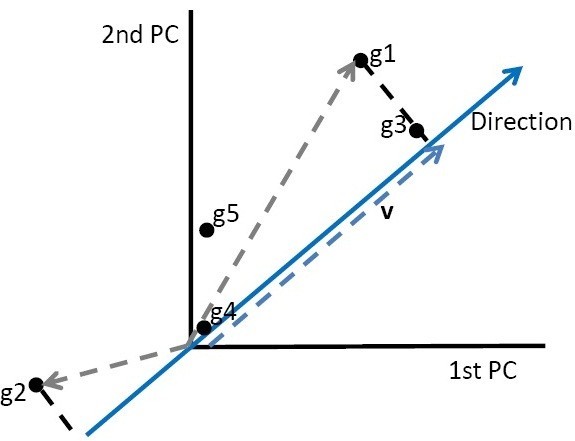
**Schematic representation of the directions and norm of the orthogonal projection of genes used to compute Cd. Gene g3 will have the highest value of Cd as its position is close to the direction and far away from the origin.** Gene g1 will have the second highest value of Cd. Gene g2 will have a negative value of Cd. Genes g4 and g5 will have values of Cd close to zero.

In order to obtain a measure that expresses the distance of a gene with respect to a direction on the principal component space (PC) in R^n^ we propose the measure C_d_. This measure will give information about how close a gene is to a given direction that represents a given biological condition for which several measures (replicates) are available. This measure is proposed based on the orthogonal projection (closest distance from a point to a line) and norm. We propose to use the projection of the gene vector on the direction in order to take account for the distance of the gene to the origin and therefore highlight genes that have an expression that differs from zero and from the mean expression behavior. Gene expression profiles containing more noise than signal will tend to be placed close to the origin and gene expression profiles that are not related to the direction of interest will be far from the direction and both cases should therefore have a low C_d_ value. Moreover, genes having expression profiles close to one direction will be far from the origin and close to the direction (Figure 1). The ratio between the norm of the projection (of the gene on the direction representing a condition) and the projected vector norm tends to 1 when the angle between the projection and the direction vector tends to 0. Basically, C_d_ will express values of 0 when a gene is far from the vector representing a given condition and 1 or −1 when a gene is close to the condition. The condition being here represented by a vector constructed as a linear combinations of all replicates of the condition of interest. Therefore, genes will be identified as belonging more to one or another condition and will be classified as behaving differentially between the biological conditions measured, based on theC_d_ closeness measure (Figure 1).

The C_d_ closeness measure is given by:

 , where  is the direction *i* with *i=1,2,..,p* (*p* being the number of directions or conditions), ∝∈R,  an indicator function that indicates if the gene belongs or not to the direction *d*_*i.*_ This means that if  for any ∝ then *,* and  otherwise*.* <>indicates the usual inner product. sign (.) returns the sign (1 or −1) of its input and .

The C_d_ closeness measure has three important zones:

: If C_d_ is equal to 0, there is no relationship with the direction and therefore no differential expression comparing both conditions. C_d_ close to one indicates a strong relationship to one condition and therefore a differential expression (activation of the gene in the condition to which it is close). This occurs when  and

: are values for which the vectors have a positive cosine to the projection on , and their value tends to 1 when the angle between then becomes shorter. On the other hand, when the angle is large, the C_d_ value will tend to 0.
: indicate values for vectors that coincide with the direction ; as higher is their norm (and they are farer away from the origin), the C_d_ closeness measure will be closer to 1.

**Figure 2:**
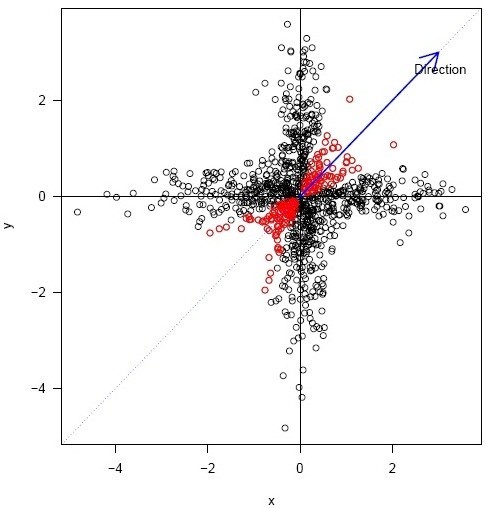
**Example of genes with Cd> 0.9 (red dots) and therefore found near the direction (blue arrow) representing one biological condition for which replicates of microarray measurements are available.**

It is also important to mention that C_d_ could take negative values when the genes are placed on the other side of the direction. This means that −1 and 1 have the same meaning, a close relationship to the direction. For an example of GENES with high C_d_ values see figure 2.

Most of the genes do not change drastically when biological conditions change. So, in order to limit the search, 25\% of genes placed in the center between conditions in the PC space are not considered for classification. Genes that have norms below the first quartile (first 25\% smallest norms) are filtered fixing their C_d_ to 0.

### False positive rate measure

Although the false discovery rate (FDR) was proposed for multiple hypothesis testing (Benjamini and Hochberg, 1995) in order to control false rejecting of null hypotheses it can also be used in the case of classification. FDR is defined by Benjamini and Hochberg (Benjamini and Hochberg 1995) as  where *V* is the number of hypotheses declared significant and that are in fact true and R is the total number of hypotheses declared significant (total hits), therefore the FDR is the expectation of *V/R* if *R* is not equal to zero and is zero otherwise.

Here, the comparison between scenarios based on simulated data is done using measures as true positives (Tp), true negatives (Tn), false positives (Fp) and false negatives (Fn) and using the , where *FP* is playing the role of *V* and *FP+Tp* is playing the role of *R* in the proper definition of the *FDR*. The classification of positive or negative hits was done using different C_d_ thresholds.

### General case simulation

**Figure 3:**
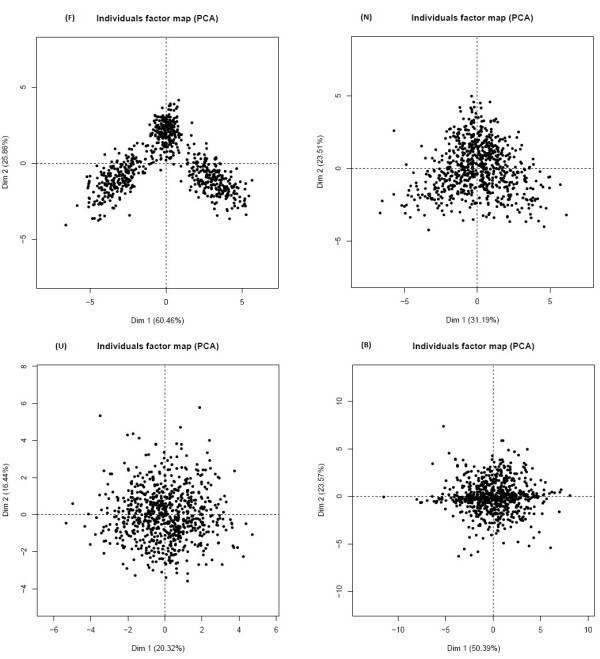
**Factorial plot of the first two PCs of simulated data starting with a favorablescenario in which genes are not confounded on the left and ending with a very bad scenario (of high confusion) on the right (Sim1).**

**Figure 4:**
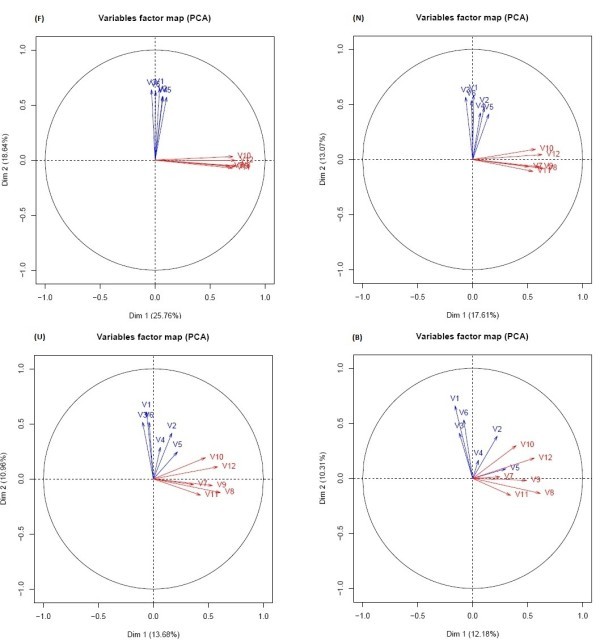
**Factorial plot of the first two PCs of simulated data starting with a favorable scenario in which conditions are not confounded on the left and ending with a very bad scenario (of high confusion) on the right (Sim2).**

Data were simulated for different case scenarios in order to investigate if C_d_ (and DAPC) are useful for detecting differentially expressed genes between conditions (here two). Data under four different scenarios (favorable (F), normal (N), unfavorable (U) and very bad (B)) were simulated. These scenarios were simulated confounding progressively individuals (genes, labeled as Sim1) and variables (replicates of biological conditions, labeled as Sim2), Figures 3 and 4. When analyzing real data both types of confusions are common: one in which genes are not structured but are, on the contrary very similar and another in which microarray conditions do not differentiate between each other. The goal of investigating performance on different scenarios was to characterize limitations of the DAPC and C_d_ closeness measure.

### Data generation

For the Sim1 data generation it was assumed that microarray data have a normal distribution (which is generally true after calibration and transformation of gene expression data). Here, microarray data belonging to two groups with differential expression were generated. So, a matrix *X* with *n*_*1*_*+n*_*2*_*=n* rows (genes) and *p*_*1*_*+p*_*2*_*=p* columns, where *n*_*1*_ and*n*_*2*_, are the number of genes belonging to each condition and *p*_*1*_ and *p*_*2*_ are the number of replicates of each condition. First, a sample from a multivariate normal distribution with mean vector 0 and correlation matrix approximately *I* is taken. The choice of the correlation matrix being exactly *I* or approximately *I* allows choosing the level of noise to be introduced in the data. Second, for the block *X*_*11*_*=X[1:n*_*1*_*,1p*_*_1*_*]* of the matrix values sampled from a multivariate normal distribution with mean vector 0 and correlation matrix W_1_ were added to the previously generated values originating distortion on the expressions for one of the two conditions (Sim1). Values sampled from a multivariate normal distribution with mean vector 0 and correlation matrix W_2_ are added in the same way to the block *X*_*22*_*=X[1:n*_*1*_*,1p*_*_1*_*]*.

For the simulation trying to confound replicates of the two conditions (Sim2) a truncated normal distribution was used to add to the replicates of the two conditions. This second method is an adaptation of the *simData* function in the R library optBiomarker (Khondoker et al., 2009) but does not differ in its methodology from the data generation used for Sim1.

The values of mean and correlation matrix for the four scenarios were chosen based on the separation of genes observed on the plot of the first two principal components (PC) when a Principal Component Analysis was conducted on the simulated data as shown in Figures 3 and 4. Sim1 is a simulation that confounds gene expression between two conditions progressively as scenarios go from favorable (F) to very bad (B) and Sim2 is a simulation that confounds replicates of both conditions progressively as scenarios go from favorable (F) to very bad (B) as shown in Figures 3 and 4.

### Identification of differentially expressed genes through classification

Here we propose four possible classifications using C_d_ and DAPC in order to classify genes into one of two conditions and therefore detect genes that are differentially expressed. The classifications we compared are the following:

- DAPC: Classification only by DAPC specifying a classification into three groups. These three groups are thought to group genes into a class of genes have similar expression across the different conditions and are clustered around the origin and two groups of genes far from the origin clustering genes into one of the two experimental conditions that are compared.
- C_d_ with DAPC: Classification by DAPC of the genes detected by C_d_ to be in a zone of interest in the PC space (C_d_>threshold). This classification is based on the groups proposed by DAPC and additionally takes into account only genes with a C_d_ higher than certain threshold to be fixed by the researcher. The values of C_d_ and therefore their thresholds can be fixed individually for each condition.
- C_d_: Classification of the genes detected by C_d_ to be close to one of the two conditions. This classification is based on the genes with C_d_ higher than a certain threshold without using the groups proposed by DAPC. Here again, the values of C_d_ and therefore their thresholds can be fixed individually for each condition.
- C_d_ inter DAPC: Intersection of genes obtained by DAPC and C_d_ separately. Here the groups are constructed with the genes that are classified independently to belong to the same biological condition by DAPC and C_d_ and then, genes classified by both methods, are used to identify the belonging to one of the two conditions.

### FDR estimation

As simulated data was used, it is possible to identify genes correctly belonging to each of the two conditions analyzed. The FDR was calculated only with the genes classified into one of the two biological conditions, which means that the genes which were classified in a third group (DAPC- labeled as ''no differential gene expression'') were not taken in to account at the moment of the FDR estimation. It is worth to point out that the FDR is often used in literature to report reliability of classification into one category only. Here it is used to report a global accuracy measure of classification in two groups. In this specific case the true positives (Tp) are the number of accurate classifications in any of the biological conditions. False positives (Fp) are the number or genes that are wrongly classified into the category ''no differential gene expression''.

### Directions representing conditions

**Figure 5:**
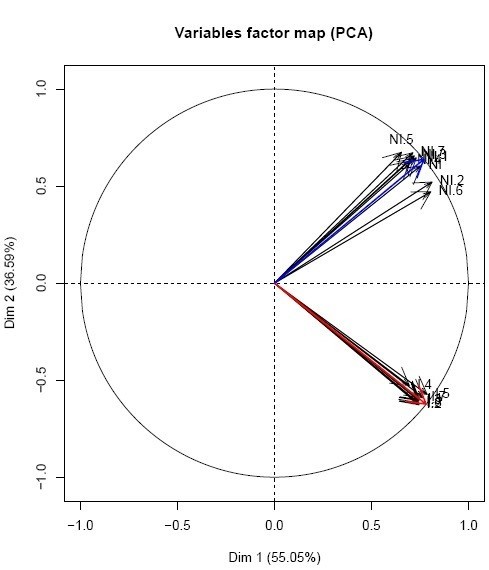
**Corcircle of a PCA conducted on the tomato microarray data set showing that replicates of the same biological conditions (Ni and I) are strongly correlated and that a mean direction representing each condition can be obtained.** Blue arrow represents Ni and red arrow represents condition.

Directions in the PC space are obtained as a mean vector of variable vectors in the PC space (Figure 4). The red arrows represent the variables (biological replicates of one condition) and blue arrows represent replicates of another condition. The mean vector of each group will represent the condition and will be used as the direction to calculate the C_d_ closeness measure. This can only be assumed if a previous PCA confirms that replicates of conditions are not confounded (Lebart et al., 1995), but are linearly correlated to each other and less correlated to replicates from the other condition. An example of directions (mean of vectors representing replicates of the same condition) is shown in Figure 5.

### C_d_ closeness measure threshold

In order to determine the best value of C_d_ for an accurate classification (based in simulated data) the following steps were undertaken.

- Selecting the scenario (F to B).
- Generating data for each combination of parameters as described before.
- Obtaining the coordinates of the genes in the PC space.
- Obtaining C_d_ and setting C_d_ =0all genes with a norm less than the 25th percentile of norms.
- For each threshold of C_d_ on starting from 0.970 to 0.999 with steps of 0.004, FDR was calculated for genes with | C_d_ |> threshold.
- Obtaining the FDR estimation as the mean of  for each set of parameters and C_d_ thresholds (0.970 to 0.999).
- Obtaining the best C_d_ threshold (maximum threshold that minimizes the FDR).
- Repeating N times to obtain a sample of FDR estimations for the whole set of parameters and best thresholds.
- Obtaining the mean of the sample for each of the parameter of interest.

As this is a mean of means it can be called a global mean or global average (avg).

- Repeat all steps for each scenario and for both simulated data sets (Sim2 and Sim1)

## Results

### Identification of differentially expressed genes in simulated data

The main purpose of this work was to obtain a differential gene detection using a multivariate method. This has been conducted as a classification of genes in the PC space and their belonging to one of two conditions. We compared and combined the here proposed closeness measure C_d_ with the previously proposed DAPC (Jombart et al., 2010) in order to evaluate accuracy of these methods based on simulated data.

**Figure 6:**
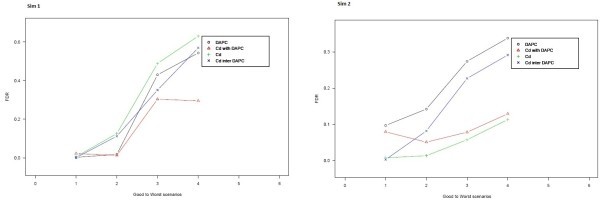
**FDR comparison plot for all four methods applied.** In the top we show resultsSim 1 (confusion of genes) scenarios and in the bottom the Sim2 (confusion of conditions) scenarios.

Simulated data allowed studying the accuracy of both methods and their combination given different degrees of gene and condition confusion. The four scenarios and four methods are explained in previous sections. For data, in which genes do not have a clear structure in separate groups (sim2), the C_d_ closeness measure posterior to a PCA is the best method, and for data in which genes form observable groups (sim1) C_d_ closeness measure with DAPC performs better. The four classifications used are compared here based on their FDR, which increases when simulation scenarios tend to unfavorable and therefore more confusion is present (Figure 6). The obtained FDR values are observable in Tables 1,2, 3,4.

**Table 1:** **FDR of each classification proposal - Sim1**

Method and scenario	Mean FDR	FDR Sd	FDR p_2.5	FDR p_50	FDR p_97.5
DAPC-favorable	0.0024	0.00276	0	0.00208	0.00834
Cd with DAPC-favorable	0.02221	0.00983	0.00455	0.02111	0.04227
Cd-favorable	0.00596	0.00138	0.00419	0.00497	0.00802
Cd inter DAPC-favorable	0.0012	0.00631	0	0	0.01638
DAPC-normal	0.01831	0.00061	0.01717	0.01829	0.01949
Cd with DAPC- normal	0.01299	0.00407	0.00757	0.01215	0.02248
Cd- normal	0.12555	0.00496	0.11787	0.12411	0.13476
Cd inter DAPC- normal	0.11197	0.11205	0.03333	0.07692	0.5
DAPC-unfavorable	0.42963	0.00144	0.42684	0.42963	0.43252
Cd with DAPC-unfavorable	0.30382	0.00779	0.28949	0.30329	0.31485
Cd-unfavorable	0.4887	0.00582	0.47921	0.49235	0.49494
Cd inter DAPC-unfavorable	0.35083	0.15161	0.11111	0.33333	0.66667
DAPC-bad	0.54284	0.00176	0.53932	0.54279	0.54624
Cd with DAPC-bad	0.29472	0.00856	0.27756	0.2939	0.31336
Cd-bad	0.62961	0.00395	0.62148	0.63069	0.63475
Cd inter DAPC-bad	0.56905	0.17942	0.2	0.57143	0.85714

**Table 2:** **FDR of each classification proposal - Sim2**

Method and scenario	Mean FDR	FDR Sd	FDR p_2.5	FDR p_50	FDR p_97.5
DAPC-favorable	0.09688	0.00314	0.09067	0.09687	0.10299
Cd with DAPC-favorable	0.07923	0.00607	0.068	0.0792	0.09132
Cd-favorable	0.00745	0.00049	0.00675	0.0075	0.00807
Cd inter DAPC-favorable	0.00257	0.00875	0	0	0.03226
DAPC-normal	0.14214	0.00352	0.13551	0.14219	0.14941
Cd with DAPC- normal	0.05079	0.00829	0.03592	0.05036	0.06696
Cd- normal	0.0133	0.00062	0.01209	0.01329	0.01456
Cd inter DAPC- normal	0.08163	0.04024	0.03704	0.06667	0.17647
DAPC-unfavorable	0.27408	0.00252	0.26896	0.27408	0.27905
Cd with DAPC-unfavorable	0.07828	0.0043	0.06844	0.07844	0.08929
Cd-unfavorable	0.05736	0.00407	0.05205	0.05484	0.06373
Cd inter DAPC-unfavorable	0.2268	0.11732	0.07143	0.2	0.5
DAPC-bad	0.33817	0.00268	0.33264	0.33829	0.34306
Cd with DAPC-bad	0.12906	0.0063	0.11663	0.12846	0.13967
Cd-bad	0.11304	0.00462	0.10167	0.11418	0.12247
Cd inter DAPC-bad	0.29145	0.14786	0.09091	0.25	0.625

**Table 3:** **Cd of each classification proposal using different Cd thresholds (1- Cd< Threshold)- Sim1**

Method and scenario	Mean threshold	Threshold Sd	Threshold p_2.5	Threshold p_50	Threshold p_97.5
Cd with DAPC-favorable	0.00504	0.00887	0.001	0.001	0.029
Cd-favorable	0.01599	0.01225	0.001	0.009	0.029
Cd with DAPC-normal	0.02865	0.00239	0.025	0.029	0.029
Cd-normal	0.00658	0.00604	0.001	0.005	0.021
Cd with DAPC-unfavorable	0.00542	0.00595	0.001	0.001	0.021
Cd-unfavorable	0.01394	0.01091	0.001	0.013	0.029
Cd with DAPC-bad	0.00244	0.00364	0.001	0.001	0.013
Cd-bad	0.02341	0.01025	0.001	0.029	0.029

**Table 4:** **Cd of each classification proposal using different Cd thresholds (1- Cd< Threshold) - Sim2**

Method and scenario	Mean threshold	Threshold Sd	Threshold p_2.5	Threshold p_50	Threshold p_97.5
Cd with DAPC-favorable	0.02898	0.00044	0.029	0.029	0.029
Cd-favorable	0.02833	0.00426	0.029	0.029	0.029
Cd with DAPC-normal	0.02881	0.00121	0.025	0.029	0.029
Cd-normal	0.02897	0.00033	0.029	0.029	0.029
Cd with DAPC-unfavorable	0.01434	0.00515	0.005	0.013	0.025
Cd-unfavorable	0.01474	0.01232	0.001	0.009	0.029
Cd with DAPC-bad	0.00723	0.00322	0.001	0.005	0.013
Cd-bad	0.01298	0.00833	0.001	0.013	0.029

These results allow us to state that, on one hand, when data shows an underlying structure that allows grouping genes (individuals) into clearly defined groups, the best method in order to obtain a reliable set of genes classified on the biological conditions is to perform DAPC and select the genes whose C_d_ closeness measure is greater than a particular threshold. In our example we show that for a threshold of 0.998, the obtained FDR is lower than 0.3 in the worst scenario of simulated data. On the other hand, when biological conditions (variables) are confounded, the best method to classify genes is using only C_d_. For a given threshold 0.987, we obtained FDR not greater than 0.12 in the worst scenario.

### Identification of differentially expressed genes in real data (tomato microarray data set)

#### PCA

First a PCA is conducted in order to determine the biological conditions that form directions on the PC space. As mentioned before, if this is not possible and replicates of biological conditions are confounded in directions, the PC space will not allow identifying directions and there for neither C_d_ nor DAPC will be suitable. For the real tomato microarray data example analyzed here, biological conditions clearly differentiate between: Not inoculated (Ni) and inoculated plants (I) (Figure 5) and therefore identifying differentially expressed genes through the here proposed methods is suitable.

#### C_d_ applied classification to the tomato dataset

Taking into account the data structure of the tomato data set (Figure 5) and the results obtained through simulation, the method that should be applied is a classification through C_d_ alone. Biological conditions are easy to represent in the factorial plane (Figure 5) and there is not a particular case of grouping structure within the genes, which set us in the case of a Sim2 normal scenario (Figure 4).

**Figure 7:**
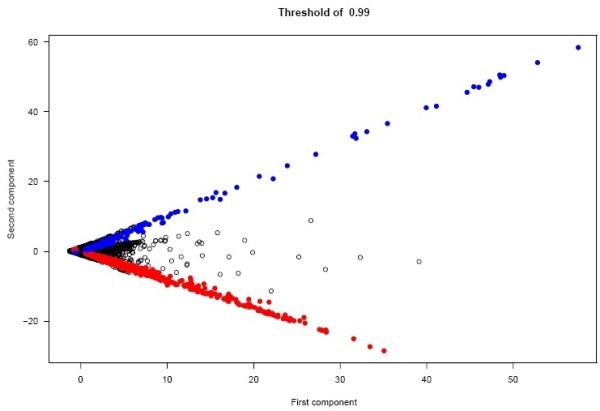
**Result of the Cd classification of the genes of the tomato data set using a threshold of 0.99.**

With this classification an FDR value no greater than 0.01456 is expected (conditions of a normal scenario). In this classification 594 genes were classified in the I group (red) and 295 genes were classified in the Ni group (Blue) among 13440 genes evaluated (Figure 7). Much more genes are classified in the I group than in the Ni group. This results are in accordance with previous results obtained analyzing this data using SAM (Tusher et al., 2001) methodology (unpublished) where for an FDR of 0.03, 734 genes were found to be upregulated at time-point 60 hours and all 594 genes identified here make part of them. No genes were found to be downregulated in this previous work. This result indicates that C_d_ is more sensitive than classical methods but also that care has to be taken with thresholds in order to avoid false positives. Genes classified by C_d_ are an indication of belonging to one or the other condition but are not a statistical proof of differential expression.

## Discussion

The proposed closeness measure C_d_ presents several advantages to classical univariate and classification methods. It allows identifying genes that belong to one or the other biological conditions and therefore have differential expression between these conditions. The fact that conditions are used as a reference for grouping, allows enhancing sensitivity for these detection. Moreover, FDRs are lower when our method is used for identification of differentially expressed genes, but this is just an indicative value, because the methodology presented here is not inferential (no P-value or FDR can be calculated for each classification). Multiple testing is avoided and a global approach allowing the detection of emerging properties due to multivariate data structure is possible with this methodology.

In general, clustering methods tend to perform their analysis over the data structure only (Lebart et al., 1995). This means that they try to create groups under distance and variance criteria (Jombart et al., 2010) among genes and groups, but do not take into account the variables, conditions or directions. Having a reference in the variable space makes a real difference in classification and proposes a precise and accurate classification. In this paper we illustrated the principal difference of using a method that classifies under data structure criteria like DAPC (Jombart et al., 2010) and a method that classifies under a direction or condition criteria(C_d_) and a combination of both. The consequence of using one method or another is shown in the FDR obtained (Tables 1 and 2), which can be very high (around 0.5 or 0.3, Figure 6) instead of 0.3 or 0.1 (for the worst scenario). The improvements are especially noticeable when biological conditions are confounded, which makes it very difficult for classical classification methods to detect differences in gene expression when comparing two conditions because no reference is present. By tuning an appropriate C_d_ threshold, it is still possible to detect differentially expressed genes even though some variables of the same condition are confounded. Moreover, the use of C_d_ alone or a combination with DAPC allows using the most appropriate method depending on data structure.

Another feature of the presented work is the possibility of reducing the amount of genes classified to the biological conditions by increasing the C_d_ threshold which would lead to groups of genes for which there is more certainty of differential expression, and moreover reduced groups of genes to perform lab proves.

Finally, we propose using the methods C_d_ with DAPC when there is a data structure, expecting in the worst case an FDR around 0.3 and C_d_ when there is not a particular data structure but there exist a sense of biological conditions or directions, expecting in the worst scenario an FDR around 0.1, for classification of biological differentially expressed genes.
